# Fear of missing out and problematic smartphone use among Chinese college students: The roles of positive and negative metacognitions about smartphone use and optimism

**DOI:** 10.1371/journal.pone.0294505

**Published:** 2023-11-28

**Authors:** Jinliang Guan, Wangyan Ma, Chengzhen Liu

**Affiliations:** 1 Department of Psychology, Hunan Normal University, Changsha, China; 2 Information Network Center, Anhui Jianzhu University, Hefei, China; 3 School of Foreign Studies, Anhui Sanlian University, Hefei, China; St John’s University, UNITED STATES

## Abstract

**Background:**

Problematic smartphone use (PSU) has become a common phenomenon among college students. Fear of missing out (FoMO) is an important factor affecting PSU, but how FoMO affects PSU is not clear. Therefore, the mediating effect of positive and negative metacognitions about smartphone use (PMSU and NMSU) and the moderating effect of optimism are explored.

**Methods:**

514 Chinese college students aged 17 to 25 from 6 Chinese universities were investigated with the Trait-State FoMO Scale, the Metacognitions about Smartphone Use Questionnaire, the Temperament Optimism Scale, and the Smartphone Addiction Scale for College Students. The data were analyzed with SPSS software.

**Results:**

FoMO was positively associated with PSU, PMSU and NMSU mediated this association. Optimism moderated the relationship between FoMO and PSU, i.e., FoMO had a less prominent positive effect on PSU for college students with a high level of optimism.

**Conclusions:**

There is a positive relationship which exists between FoMO and PSU among college students. In addition, PMSU and NMSU play mediating roles in FoMO and PSU, and optimism plays an moderating role in FoMO and PSU. These findings can help not only educators understand the predictors of PSU and develop interventions to effectively prevent PSU among college students but also college students reduce the level of PSU by improving their understanding of PMSU and NMSU and optimism level.

## Introduction

Some studies have suggested that problematic smartphone use (PSU) refers to an unhealthy pattern of mobile phone use that can impair users’ daily functions [1–3]. Billieux argues that PSU refers to uncontrolled or excessive use of mobile phones that has an impact on daily life [4]. Shin and Dey defined PSU from the perspective of behavioral consequences, referring to the situation in which an individual overuses a smartphone, which leads to impulsive smartphone use and indifference to the surrounding environment [5]. This study refers to the definition of Liu et al. [6], and defines PSU as a kind of use behavior in which an individual’s social function is impaired due to excessive use of smartphones and the inability to control such use behavior, resulting in a series of psychological and behavioral problems. The scale measuring smartphone addiction is still the most common measure [7]. Due to growing up with a new generation of cutting-edge technology, current college students are particularly inclined to embrace innovative tools like smartphones for communication and entertainment purposes [8]. However, excessive smartphone use among young people may negatively impact their life satisfaction, happiness levels, and subjective well-being [9]. Additionally, it can result in physiological issues such as headaches and poor sleep quality [10–12].

Fear of missing out (FoMO) refers to "a common fear that beneficial experiences may be obtained by others because of one’s absence" [13]. FoMO is composed of two parts: the fear of missing out on something interesting that someone else is experiencing, and the constant desire to keep in touch with friends and relatives on social networks. The former involves cognitive aspects of worry, while the latter is a behavioral strategy that includes frequent information checking, maintaining social connections, and avoiding the fear of missing out on beneficial experiences [13]. The behavior of constantly checking online information caused by FoMO makes smartphones common social tools [14]. However, it can also have adverse effects such as reduced concentration and disruptions in daily work, study, and life, making it challenging to complete tasks effectively [15–17]. A research has shown a high incidence of problematic smartphone use among college students [18]. Therefore, it is important to explore the influence of FoMO on PSU among college students to promote their physical and mental health development.

Studies have shown that FoMO is an important factor affecting PSU [19–22]. However, the precise mechanism by which FoMO influences PSU remains unclear. A precise understanding of how FoMO affects PSU can inform effective interventions for PSU. Therefore, this study aims to explore how FoMO affects PSU and identify circumstances in which this effect becomes stronger or weaker.

### FoMO and PSU

A previous study has shown that FoMO can directly or indirectly influence phubbing behavior through problematic Instagram usage [23]. Specifically, it has been found that FoMO may be a significant source of negative or depressed emotions [24]. Another previous study has demonstrated that PSU serves as a coping mechanism to alleviate negative emotions caused by stress in unfavorable living environments. Furthermore, anxiety and high perceived stress have been identified as risk factors for PSU [25]. A study has shown that students with FoMO are more likely to interrupt their daily activities to attend to smartphone notifications, which can lead to the adoption of less efficient learning strategies. Instant notifications (INs) have the potential to disrupt a smartphone user’s attention while performing tasks or engaging in activities by grabbing their attention with updates or actions. According to the Threaded Cognitive Model [26], when cognitive resources are allocated to learning-related tasks, INs that compete for cognitive resources may interfere with and hinder task performance. When individuals are drawn towards IN content, they may adopt a surface learning approach as a compensation for reduced cognitive resources [27]. Therefore, PSU may serve as a coping mechanism for dealing with FoMO [28].

In addition, self-determination theory (SDT) holds that intrinsic motivation refers to the tendency of individuals to seek new experiences, explore, and learn without external rewards [29]. Intrinsic motivation is promoted when individuals’ intrinsic need for socialized interpersonal relationships is satisfied. However, individuals experiencing FoMO may struggle to promote their intrinsic motivation if their internal needs are not met, resulting in a state of aversion to negative emotions [30]. To fulfill their internal needs, individuals experiencing FoMO may turn to the internet and social networks to acquire social skills [13]. Consequently, individuals with higher levels of FoMO may be prone to overusing the internet and social networks, leading to problematic internet use. For example, a study conducted by Elhai et al. revealed a moderate positive correlation between FoMO and smartphone addiction, with FoMO indirectly predicting social media addiction through PSU [20, 31].

Studies have shown that FoMO is related to the severity of PSU [19–22]. In this study, the relationship between FoMO and PSU is verified among Chinese college students. How FoMO affects PSU and when this effect becomes stronger or weaker are further discussed.

### The mediating roles of positive and negative metacognitions about smartphone use (PMSU and NMSU)

Metacognitions about smartphone use refer to specific beliefs generated by individuals’ smartphone usage behavior, which can be categorized into two types: positive metacognitions about smartphone use (PMSU) (e.g., ’Using my smartphone reduces my negative feelings’) and negative metacognitions about smartphone use (NMSU) (e.g., ’Using my smartphone will harm my mind’) [32]. Among these, positive metacognitions are activated before the initiation of the behavior, aiming to motivate individuals to engage in addictive behavior. On the other hand, negative metacognitions are activated during and after the occurrence of the behavior, focusing on the perpetuation of addictive behavior [33]. A study conducted by Shi et al. demonstrated that specific behavioral metacognition can predict the PSU of college students [32].

However, as college students gradually realize the uncontrollable nature of their smartphone use and its negative impact (e.g., ’Smartphone use has affected my daily life’), such realizations can trigger rumination about this behavior and its adverse consequences. This, in turn, can lead to increased anxiety. Consequently, college students may continue using their smartphones to regulate their imbalanced internal state in response to the impact of such realizations [34]. Negative metacognitions about the uncontrollability of smartphone use and cognitive harm can predict the persistence and perpetuation of PSU [35]. Specifically, in metacognitive models of psychopathology [36], positive metacognitions are believed to drive the activation of coping strategies such as worry and rumination, which can backfire and lead to the escalation of negative effects. This, in turn, activates negative metacognitions associated with the uncontrollability and dangers of escalating negative effects, trapping individuals in a state of constant psychological distress. As addictive behaviors develop and worsen over time, negative metacognitions, particularly beliefs about the uncontrollability of addictive behaviors, become the most significant predictor of maintenance and continuation [37]. Both PMSU and NMSU can contribute to PSU [33, 38]. Additionally, a study in the field of gambling has shown that the link between positive metacognitions and the severity of addiction is strong among non-clinical gamblers, while negative metacognitions play crucial roles in predicting the severity of addiction among clinical gamblers [39].

In addition, according to the model of self-regulated executive function (S-REF), worry is associated with specific metacognitions. Worry can lead to reduced cognitive function efficiency and attentional bias [40]. A previous study has indicated that positive metacognitions can reduce the anxiety caused by FoMO [41]. In addition, individuals tend to develop negative metacognitive beliefs when they experience anxiety [42]. That is, negative metacognitive beliefs about uncontrollability and danger are more pronounced in individuals with psychological distress compared to healthy individuals [40]. Since FoMO may give rise to negative or depressed emotions [24], FoMO may contribute to the development of PMSU and NMSU.

A previous study has shown that FoMO is associated with psychological distress among individuals [13]. Furthermore, positive metacognitions have been found to mediate the link between emotional regulation and problematic Internet use [43]. Another study has suggested that negative metacognition mediates the relationship between psychological distress and addictive behaviors, such as problematic internet use [37, 44]. For example, a recent study found that negative metacognitions mediate the relationship between psychological distress and PSU in an online survey of 535 smartphone users aged 18–65 [45]. However, few prior studies have examined the mediating roles of PMSU and NMSU in the relationship between FoMO and PSU. Therefore, this study aims to explore the mediating effect of PMSU and NMSU on the relationship between FoMO and PSU.

### The moderating role of optimism

Temperamental optimism is a stable personality trait that refers to an individual’s hopeful outlook for a better future, which can serve as a protective factor against risks [46]. Individuals with high social anxiety often turn to the internet to compensate for the lack of social activities and support in real life. Conversely, optimists are more likely to adopt positive problem-solving strategies to cope with loneliness, reducing the likelihood of excessive internet use [47, 48]. The attribution style theory of optimism suggests that individuals with different levels of optimism perceive information processing differently [49]. Those with high levels of optimism tend to view negative information as temporary and external factors. When encountering social networking site content favoring others, optimists interpret it as a temporary state and maintain positive expectations for the future. They motivate themselves to achieve similar outcomes, thereby mitigating the negative effects of FoMO and reducing mobile phone addiction. Studies have shown that optimists experience a faster increase in social support than pessimists when dealing with negative emotions in college [50]. Increased social support contributes to a decrease in the likelihood of mobile phone dependence [51]. A study has demonstrated that psychological resilience moderates the relationship between FoMO and PSU during epidemics [52]. This may be because individuals with high resilience tend to maintain more stable levels of emotional and mental health, resulting in lower PSU [53, 54]. Optimism, as an important component of psychological resilience, may also play a moderating role in the relationship between FoMO and PSU.

### The current study

**Fig 1:**
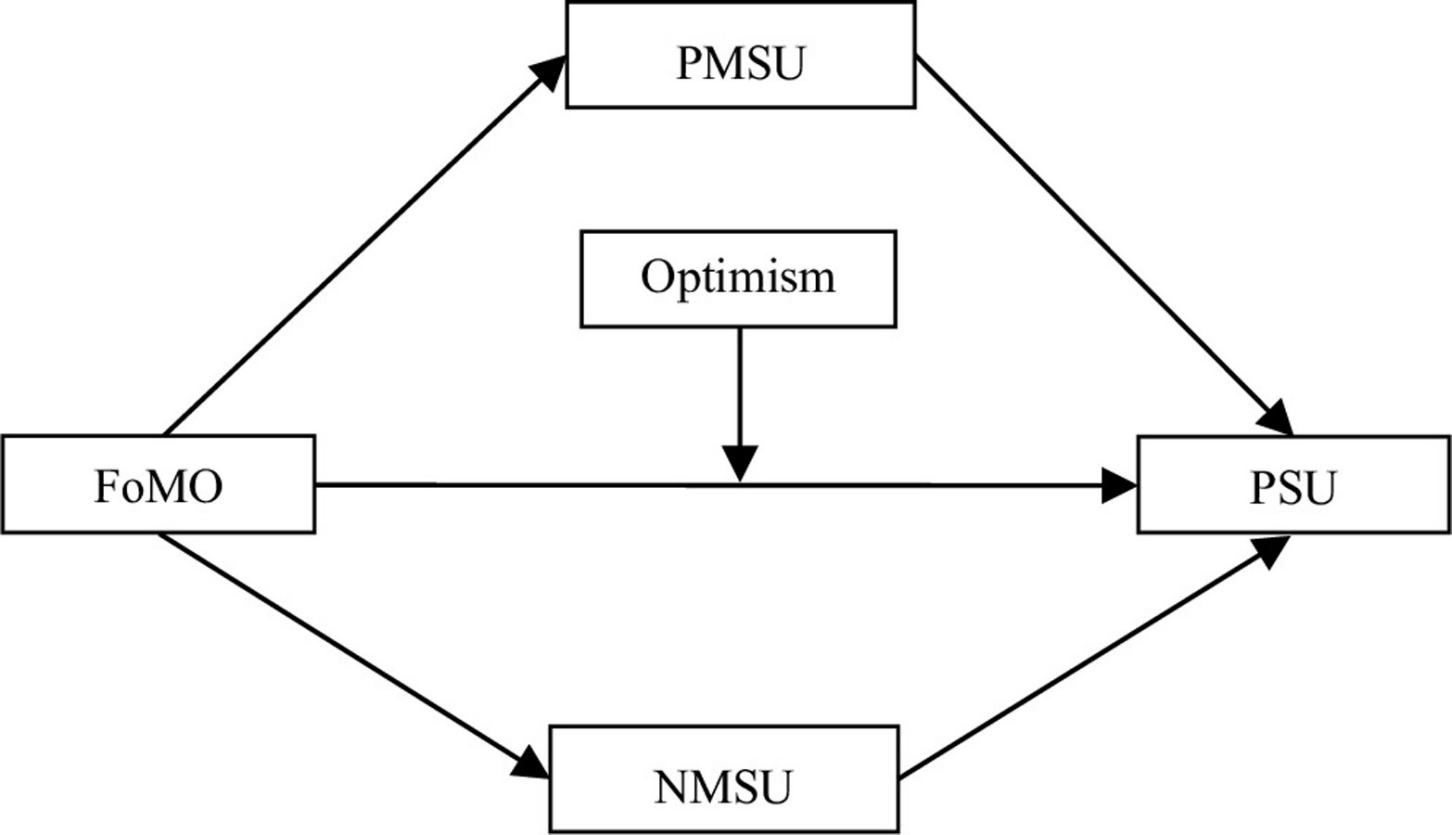
The hypothetical model. Note: FoMO = Fear of missing out, PMSU = Positive metacognitions about smartphone use, NMSU = Negative metacognitions about smartphone use, PSU = Problematic smartphone use.

Previous studies have shown that FoMO is a key predictor of PSU [19–22]. However, how FoMO affects PSU is not clear. The researchers propose a theoretical hypothesis model (Fig 1), in which the mediating role of NMSU and the moderating role of optimism are tested. The model deepens the direct relationship between FoMO and PSU, thereby responding not only how FoMO affects PSU, but also how this effect changes. Hypotheses are as the following:

#### Hypothesis 1

FoMO is positively related to PSU. Self-determination theory (SDT) holds that people tend to actively seek new experiences, explore, and learn in the absence of external rewards [29]. When individuals’ intrinsic needs for social relationships are met, their intrinsic motivation is enhanced. However, individuals experiencing FoMO, when their internal needs are not met, are unable to stimulate intrinsic motivation and instead fall into a state of aversion to negative emotions [30]. To fulfill their intrinsic needs, these individuals may turn to the Internet and social networks to upgrade their social skills [13]. Consequently, individuals who experience more severe FoMO may develop excessive reliance on the Internet and social networks, resulting in problematic Internet usage.

#### Hypothesis 2

PMSU and NMSU mediate the relationship between FoMO and PSU. The self-regulated executive function (S-REF) model suggests that anxiety is associated with specific metacognitions. A previous study has indicated that positive metacognitions can reduce anxiety caused by FoMO [41]. In addition, individuals tend to develop negative metacognitive beliefs when they experience anxiety [42]. That is, negative metacognitive beliefs about uncontrollability and danger are more pronounced in individuals with psychological distress compared to healthy individuals [40]. Since FoMO can contribute to negative or depressed moods [24], it is plausible that FoMO may lead to the development of PMSU and NMSU. Furthermore, both PMSU and NMSU can contribute to PSU [33, 38].

#### Hypothesis 3

Optimism moderates the relationship between FoMO and PSU. Given the potential lack of social support from others in real life, individuals with high social anxiety often compensate by engaging in online networking activities. Conversely, optimists are more inclined to utilize positive problem-solving strategies to cope with loneliness, thereby reducing the likelihood of excessive Internet use [47, 48].

## Materials and methods

### Participants

Since this study explored the relationship between FoMO and PSU among Chinese college students, the participants in this study were Chinese college students. In order to obtain a broad sample, 531 Chinese college students from 6 Chinese universities in Hunan Province, Anhui Province, Sichuan Province and Guangxi Zhuang Autonomous Region were selected as participants. Each participant was with a previous smartphone experience. The data were obtained by offline approaches. Offline data were collected by convenience sampling. This investigation was approved by the Biomedical Research Ethics Committee of Hunan Normal University. Written informed consent was obtained from the participants. A well-trained postgraduate introduced the principles of voluntary participation and confidentiality to the students before they filled in the questionnaire. The students completed the questionnaire after class. Each student got a small gift as a reward.

According to Presser et al., the ratio of sample size to questionnaire items should be at least 10:1 [55]. There were 39 questionnaire items in this study, and the sample size should be at least 390. According to this standard, the number of participants in this study was 514, which met the requirements of psychometric measurement. A total of 531 college students participated in the survey and filled out the questionnaires. 17 invalid questionnaires were excluded, including 3 with serious lack of data, 6 with regular answers, and 8 with scores of more than three standard deviations. The number of valid questionnaires was 514 with an effective rate of 96.80%. 224 students were male (43.58%) and 290 students were female (56.42%). There were 173 freshmen (33.66%), 162 sophomores (31.52%), 129 juniors (30.93%) and 50 seniors (9.73%), from 17 to 25 years old (*M* = 19.93; *SD* = 1.37).

### Measures

#### Smartphone Addiction Scale for College Students (SAS-C)

SAS-C, compiled by Su, Pan et al. (2014 Chinese version) contains 22 questions (e.g., "I feel the need to spend more time on my phones to be satisfied") with six dimensions of highlighting behavior, social comfort, withdrawal behavior, app updates, app use and negative influence [56]. A 5-point scale is used (1 = strongly disagree, 5 = strongly agree). A previous study has shown that this scale has good reliability and validity among Chinese college students [56]. The higher score means the higher level of problematic smartphone use. The Cronbach’s α in this study is 0.91.

#### Trait-State Fear of Missing Out Scale (T-S FoMOS)

T-S FoMOS is developed by Wegmann et al. (2017 English version) and revised by Xiao and Liu (2019 Chinese version) in the self-report scale among Chinese college students (e.g., "I am worried that my friend has more rewarding experiences than I do") [57, 58]. There are two dimensions, namely, trait-FoMO and state-FoMO. A 5-point scale is used (1 = strongly disagree, 5 = strongly agree). A previous study has shown that this scale has good reliability and validity among Chinese college students [58]. The higher score indicates the more serious fear of missing out. The Cronbach’s α of T-S FoMOS in this study is 0.86. The Cronbach’s α of trait-FoMO dimension is 0.85, and the Cronbach’s α of state-FoMO dimension is 0.80.

#### Chinese metacognitions about smartphone use Questionnaire (Chinese MSUQ)

This questionnaire, compiled by Casale et al. (2020 English version) and revised by Shi et al. (2021 Chinese version), is adopted [32, 59]. It has 24 questions in total (e.g., "I have no control over how much time I use my smartphone"), which are grouped into two dimensions, namely, positive metacognitions concerning emotional and cognitive regulation and social advantages of smartphone use (MSUQ-PM) and negative metacognitions about uncontrollability and cognitive harm of smartphone use (MSUQ-NM). A 4-point scale is used (1 = disagree, 4 = strongly agree). A previous study has shown that this questionnaire has good reliability and validity among Chinese college students [32]. In this study, the Cronbach’s α of MSUQ-PM is 0.92, and the Cronbach’s α of MSUQ-NM is 0.85.

#### Temperamental optimism scale (LOT)

This scale, compiled by Scheier and Carver (1985 English version) and revised by Wen (2012 Chinese version), has 6 questions altogether (e.g., "I am optimistic about my future") with two dimensions of the optimism factor and pessimism factor [46, 60]. A 5-point scale is used (1 = strongly disagree, 5 = strongly agree). A previous study has shown that this scale has good reliability and validity among Chinese college students [61]. Among the 6 questions, 1, 3 and 6 are positive lexical questions, while 2, 4 and 5 are negative lexical questions. The scores of the three negative lexical questions are scored in reverse and then added with the scores of the three positive lexical questions as the total score of the optimism scale. A higher score indicates a higher level of temperamental optimism. In this study the Cronbach’s α value is 0.74.

### Data analysis

Data were analyzed with SPSS version 26.0. First, descriptive statistics and Pearson correlation between variables were calculated. Second, Model 4 and Model 5 in the PROCESS macro were used to test the mediating effect of NMSU and the moderating effect of optimism.

## Results

### Common method bias

The common method bias of the data was tested by Harman’s single-factor model. Principal component analysis was conducted for all variables at the same time, and the results showed that there were 12 factors with eigenvalues greater than 1. The variance explained by the first factor was 15.00%, less than the critical standard of 40%. This result indicated that there was no serious common method bias in the data.

### Current state of PSU among Chinese college students

**Fig 2:**
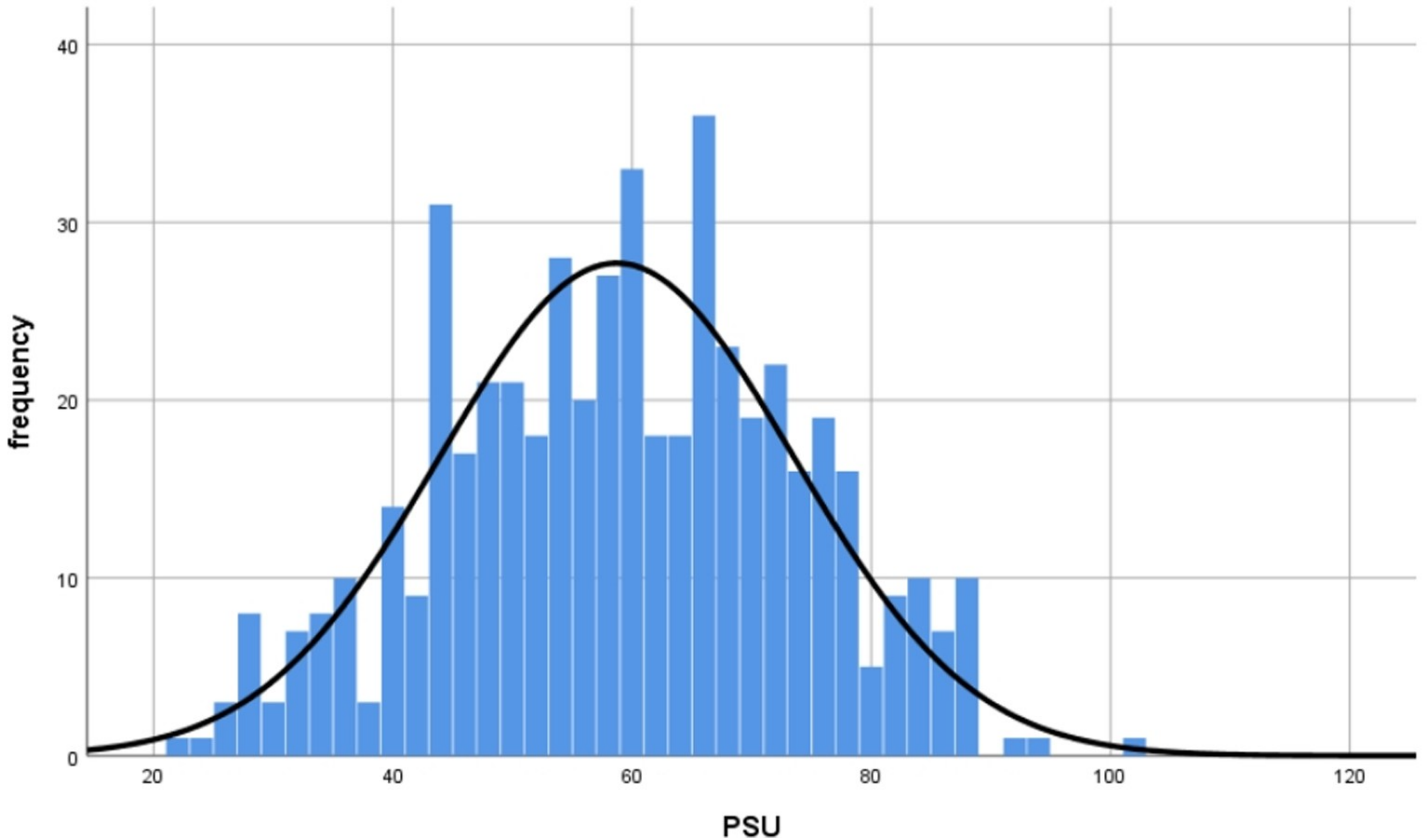
The score distribution histogram of PSU.

According to the classification standard of PSU by Su et al., there were 60 college students with PSU scores of more than 77 points, and they are students with high PSU levels [62]. The score distribution histogram of PSU is shown in Fig 2.

### Descriptive statistics and correlation analysis

In Table 1, correlation analysis showed that FoMO was positively correlated with PMSU, NMSU and PSU. FoMO was negatively related to optimism. PMSU and NMSU were positively related to PSU. NMSU were negatively related to optimism. PSU was negatively related to optimism. The independent sample *t* test was used to examine the gender differences between FoMO and PSU. The results showed that there was no significant difference in FoMO (*t*(512) = -0.27, *p* = 0.79) and PSU (*t*(512) = -0.81, *p* = 0.42) between genders. In addition, it can be seen from Table 2 that age has no significant correlation with FoMO and PSU.

**Table 1:** Means, standard deviations and correlations of the variables (*N* = 514).

	*M*	*SD*	1	2	3	4	5	6
1 Gender	1.56	0.50	1					
2 Age	19.93	1.37	-0.01	1				
3 FoMO	28.01	7.70	0.01	0.03	1			
4 PMSU	33.04	8.37	-0.01	-0.01	0.15^**^	1		
5 NMSU	18.97	5.48	0.00	0.08	0.56^***^	0.23^***^	1	
6 PSU	58.71	14.80	0.04	0.07	0.67^***^	0.25^***^	0.73^***^	1
7 Optimism	21.57	3.91	0.15^**^	-0.08	-0.40^***^	0.06	-0.40^***^	-0.41^***^

Note: FoMO = Fear of missing out, PSU = Problematic smartphone use, PMSU = Positive metacognitions about smartphone use; NMSU = Negative metacognitions about smartphone use

^**^p < 0.01

^***^p < 0.001.

**Table 2:** The fit statistic of mediating effect.

Variables	Equation 1: PSU				Equation 2: PMSU				Equation 3: NMSU				Equation 4: PSU			
	*B*	*Β*	*SE*	*t*	*B*	*β*	*SE*	*t*	*B*	*β*	*SE*	*t*	*B*	*β*	*SE*	*t*
FoMO	1.29	0.67	0.03	20.46^***^	0.16	0.15	0.04	3.35^***^	0.40	0.56	0.04	15.39^***^	0.72	0.38	0.03	11.73^***^
PMSU													0.14	0.08	0.03	2.84^**^
NMSU													1.36	0.50	0.03	15.30^**^^*^
Gender	0.86	0.06	0.07	0.88	-0.11	-0.01	0.09	-0.15	-0.07	-0.01	0.07	-0.17	0.97	0.07	0.05	1.22
Age	0.52	0.04	0.02	1.48	-0.10	-0.01	0.03	-0.36	0.26	0.05	0.03	1.79	0.18	0.01	0.02	0.63
*R*	0.67				0.15				0.57				0.80			
*R* ^*2*^	0.45				0.02				0.32				0.64			
*F*	141.47^***^				3.76^*^				80.59^***^				182.39^***^			
*df1*	3				3				3				5			
*df2*	510				510				510				508			

Note: FoMO = Fear of missing out, PSU = Problematic smartphone use, PMSU = Positive metacognitions about smartphone use; NMSU = Negative metacognitions about smartphone use;

^*^p < 0.05,

^**^p < 0.01,

^***^p < 0.001.

### Testing for mediation

Some related studies have shown that gender and age have an important impact on individual internet use [63, 64]. Therefore, these two variables were included as control variables in the data analysis.

The total effect of FoMO on PSU was positive. With PMSU and NMSU as the mediator, FoMO positively predicted PMSU and NMSU, PMSU and NMSU significantly positively predicted PSU. The results indicated that PMSU and NMSU played a partially mediating role in the influence of FoMO on PSU (Table 2). The mediating effect size of PMSU accounted for 1.70% of the total effect. The mediating effect size of NMSU accounted for 42.03% of the total effect.

### Testing for moderation

In order to test the moderating effect of optimism, the PROCESS macro was used. Gender and age factors were controlled. The results were as follows (Table 3). In Equation 1, FoMO significantly positively predicted PMSU. In Equation 2, FoMO significantly positively predicted NMSU. In Equation 3, FoMO significantly positively predicted PSU. The interaction term between FoMO and optimism significantly predicted PSU. The results of the model estimation verified that the relationship between FoMO and PSU was moderated by optimism.

**Table 3:** The moderating effect of optimism.

Variables	Equation 1: PMSU				Equation 2: NMSU				Equation 3: PSU			
	*B*	*β*	*SE*	*t*	*B*	*β*	*SE*	*t*	*B*	*β*	*SE*	*t*
FoMO	0.16	0.15	0.04	3.35^***^	0.40	0.56	0.04	15.39^***^	1.32	0.35	0.03	10.77^***^
Optimism									-0.02	-0.09	0.03	-3.12^**^
FoMO×Optimism									-0.03	-0.06	0.02	-2.48^*^
PMSU									0.16	0.09	0.03	3.37^***^
NMSU									1.27	0.47	0.03	13.96^***^
Gender	-0.11	-0.01	0.09	-0.15	-0.07	-0.01	0.07	-0.17	1.42	0.10	0.05	1.79
Age	-0.10	-0.01	0.03	-0.36	0.26	0.05	0.03	1.79	0.16	0.01	0.02	0.55
*R*	0.15				0.57				0.81			
*R* ^*2*^	0.02				0.32				0.65			
*F*	3.76^*^				80.59^***^				136.14^***^			
*df1*	3				3				7			
*df2*	510				510				506			

Note: FoMO = Fear of missing out, PSU = Problematic smartphone use, PMSU = Positive metacognitions about smartphone use; NMSU = Negative metacognitions about smartphone use;

^*^p < 0.05,

^**^p < 0.01,

^***^p < 0.001.

**Fig 3:**
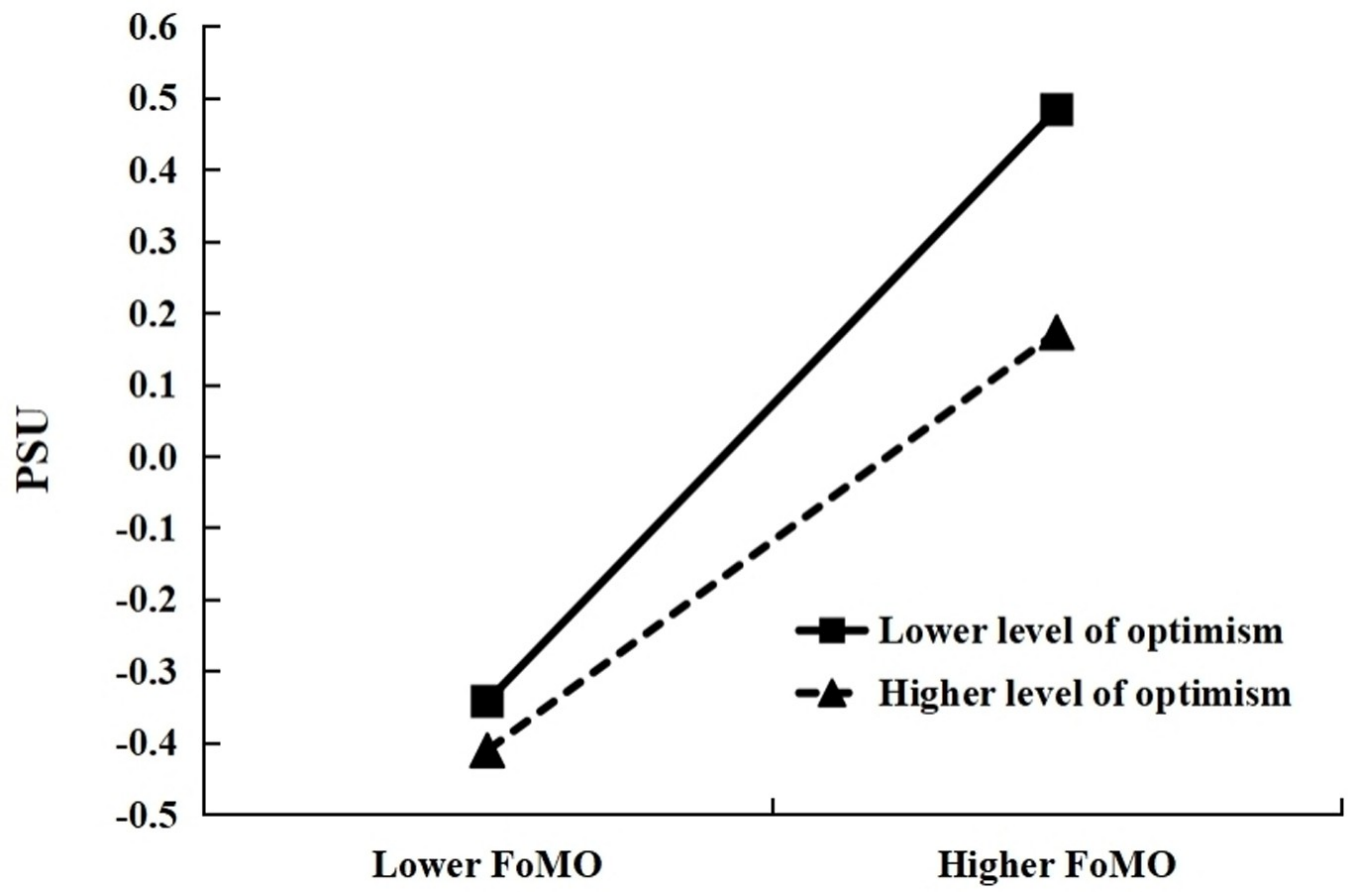
The moderating effect of optimism on the relationship between FoMO and PSU. Note: FoMO = Fear of missing out, PSU = Problematic smartphone use.

Furthermore, the relationship between FoMO and PSU at two different levels of optimism (M + 1 SD and M– 1 SD) was presented in Fig 3. The results showed that FoMO positively predicted PSU at the lower level (M– 1 SD) (*β*_simple_ = 0.41, *t* = 10.06, *p* < 0.001, 95%CI: 0.33 to 0.49), while FoMO had a weaker positive predictive effect on PSU at the higher level (M + 1 SD) (*β*_simple_ = 0.29, *t* = 7.19, *p* < 0.001, 95%CI: 0.21 to 0.37). The results indicated that the higher the level of optimism was, the lower the impact of FoMO on PSU was.

## Discussion

### FoMO and PSU

The findings of this study indicate that the percentage of college students with high PSU levels is 11.67%, which is consistent with a previous study [65]. This study has demonstrated that FoMO significantly and positively predicts PSU among college students, thus, Hypothesis 1 is accepted. Specifically, FoMO may contribute to negative or depressed emotions [24], leading individuals to excessively use electronic devices as a means to regulate these negative emotional experiences. FoMO can serve as a predictor of the severity of PSU, as the unsatisfied need for social relationships caused by FoMO drives individuals to use their phones more frequently to check messages. Furthermore, the habitual use of smartphones can escalate into excessive use [66].

Our findings support previous research that suggests a relationship between FoMO and smartphone overuse [21, 67]. Individuals with high levels of FoMO are likely to experience difficulties with smartphone use and emotional regulation [28]. FoMO refers to the need to stay updated on other people’s activities in social life and the fear of not being able to keep up with trends [13]. People read positive emotional content online and assume that their friends are still enjoying their lives when they are offline [68]. Furthermore, individuals’ FoMO and excessive internet use have a negative relationship with their level of subjective well-being [69], which can be seen as one of the adverse effects of their fear of not being able to keep up with social life’s developments.

### The mediating roles of PMSU and NMSU

Another finding of this study is that PMSU and NMSU partially mediate the relationship between FoMO and PSU among college students, providing support for Hypothesis 2. According to the self-regulated executive function model, a specific response pattern known as the cognition-attention syndrome can trap individuals in long-term emotional experiences and conflicts in self-regulation. These conflicts can lead to feelings of helplessness and a loss of adaptive control over cognition and emotion, resulting in mental health problems [36]. This result supports a previous study suggesting that PMSU occur before mobile phone use behaviors and is designed to motivate individuals to engage in addictive behaviors, while NMSU occur during and after mobile phone use behaviors, triggering the escalation of psychological distress [33]. This further compels college students to continue using smartphones as a means to regulate their internal state, ultimately leading to PSU [34].

This study confirms a previous study that has found an association between FoMO and metacognitions [41]. A study has shown that positive metacognitions mediate the link between emotional regulation and problematic Internet use [43]. Therefore, the role of positive metacognitions in emotional regulation may lead to the occurrence of addictive behaviors. A study has demonstrated that negative metacognitions mediate the relationship between negative emotions and technology addiction [70]. The identification of specific negative metacognitions associated with behavioral addictions can help elucidate the pathways involved in the development of these addictive behaviors. This study is consistent with a previous research suggesting that the presence of specific metacognitions (such as PMSU and NMSU) lead to specific behavioral addictions (such as mobile phone addiction) [71]. This result supports the roles of PMSU and NMSU in addictive behaviors, indicating that positive metacognitions stimulate the occurrence of addictive behaviors, while negative metacognitions play a critical role in the perpetuation of addictive behaviors [37].

### The moderating role of optimism

The results have shown that optimism moderates the relationship between FoMO and PSU. Specifically, in the current study, FoMO exerts a significantly positive predictive effect on PSU for those with a low level of optimism, while FoMO has a weaker positive predictive effect on PSU for those with a high level of optimism. Thus, the results support Hypothesis 3. Optimism, as a positive personality trait, acts as a protective factor promoting social adaptation and serves as a cushioning factor against risks.

Based on the scores from the optimism scale, high optimists tend to score higher on the optimism dimension and lower on the pessimism dimension, indicating that high optimists exhibit the characteristics of high optimism and low pessimism. This finding aligns with the results of a previous study, which suggest that high levels of optimism and low levels of pessimism represent the optimal temperamental optimism [72]. Individuals who possess both high levels of optimism and low levels of pessimism tend to experience high levels of subjective well-being [72]. Scheier et al. proposed that temperamental optimism refers to an individual’s overall expectation for positive outcomes in the future. It is considered a stable personality trait with high stability [73]. Optimistic individuals have positive expectations about future events and believe in the likelihood of positive outcomes. As such, optimism serves as an important internal resource for regulating the physical and mental health of individuals [74].

First, the results support a previous study indicating that optimists are more inclined to employ positive problem-solving strategies to cope with feelings of loneliness [47]. Second, this study provides support for the motivation theory of optimism. According to this theory, individuals with optimistic personalities are more likely to adopt problem-solving coping strategies, which enhance their ability and skills in managing social relationship problems and help prevent internet addiction [75]. Third, this study supports the attribution style theory of optimism. Individuals with high levels of optimism tend to perceive adverse information as external factors and are more inclined to apply coping strategies to address their own problems. This positive mindset influences their attitude towards existing social networks [47]. This study validates previous studies, demonstrating that individuals with high resilience exhibit more stable levels of emotional and mental health and have a lower likelihood of experiencing PSU [53, 54].

### Limitations, future directions, and contributions

There are several limitations in this study that may guide future research directions. First, this study is cross-sectional in nature, limiting its ability to establish causal relationships. Future researchers should consider longitudinal studies, such as employing cross-hysteresis models, to investigate causal associations between variables. Second, college students exhibit developmental characteristics that vary across different stages of their university education [76]. The relationship between FoMO and PSU may change as students progress through different grade levels. Future researchers can explore the developmental aspects of FoMO and its impact on PSU. Third, the universities included in this study are predominantly located in the southern and western regions of China. To enhance generalizability, future studies should aim to broaden the selection range of universities to encompass a more representative sample from all over China, and increase the sample size accordingly. Last, this study relied on self-reporting as a means to assess PSU. However, recent researches have indicated that employing objective measures to evaluate smartphone use offers numerous advantages over self-reporting, primarily due to the enhanced reliability associated with objective measurements [77–79]. Consequently, future endeavors should strive to utilize objective measurement techniques to assess PSU levels, thereby yielding more objective and accurate outcomes.

Despite these limitations, this study has some theoretical and practical significance. In terms of theoretical significance, first, it aligns with the self-determination theory (SDT), which suggests that individuals turn to the Internet and social networks to enhance their social skills when their intrinsic needs are not being fulfilled. Second, the findings align with the self-regulatory executive function model (S-REF), indicating that anxiety is linked to specific metacognitive processes, leading to reduced cognitive efficiency and attention bias. Last, the study supports the theory of positive motivation, highlighting the self-protective role of a positive and optimistic attitude. Such an attitude can help individuals relieve the anxiety caused by FoMO and decrease the likelihood of excessive Internet usage. In terms of practical significance, first, the results of this study verify the important impact of FoMO on the PSU of college students. Therefore, it is necessary to decrease the sensitivity of college students to FoMO, including physical exercise and mindfulness meditation, which can lower the anxiety caused by FoMO to a certain extent. Second, college students in the transition stage need to improve their understanding of the positive and negative metacognitions related to smartphone use and minimize the harm caused by PMSU and NMSU. Third, administrations should be concerned about the mental health education of college students, guide students to adjust their attitude in time, deal with the anxiety caused by FoMO with an optimistic attitude, and communicate with counselors and teachers in time to lower the tendency of PSU. Last, to reduce their reliance on smartphones, college students are encouraged to establish learning objectives, create sensible study schedules, and actively engage in school-organized activities. Additionally, active participation in various school activities fosters a sense of belonging and promotes alternative avenues for social interaction, reducing the need for constant smartphone use.

## Conclusions

The researchers explored the impact mechanism of the effect of FoMO on PSU among Chinese college students. The main aim of this study was to explore the mediating effect of PMSU and NMSU and the moderating effect of optimism. By collecting data with a questionnaire survey of 514 college students, the researchers found that (1) FoMO positively predicted PSU; (2) PMSU and NMSU mediated the relationship between FoMO and PSU; (3) optimism moderated the relationship between FoMO and PSU, i.e., FoMO had a less prominent positive effect on PSU for college students with a high level of optimism. These findings can help not only educators understand the predictors of PSU and develop interventions to effectively prevent PSU among college students but also college students reduce the level of PSU by improving their understanding of PMSU and NMSU and optimism level.

## Supporting information

S1 File Questionnaires about college students’ smartphone use. (DOCX)
