# Supplementary material for: Fear of missing out and problematic smartphone use among Chinese college students: The roles of positive and negative metacognitions about smartphone use and optimism
Source: PLoS One. 2023 Nov 28;18(11):e0294505. doi: 10.1371/journal.pone.0294505 (PMC10684080; doi:10.1371/journal.pone.0294505)
Supplement: S1 File — (DOCX) [file pone.0294505.s001.docx]

Questionnaires about College students’ smartphone use

一、Basic situation

| 1.Age：□16 □17 □18 □19 □20 □21 □22 □23 □24 □25  2.Gender：□Male □Female  3.Category of specialty：□Liberal arts □Science and engineering  4.Grade：□Freshman □Sophomore □Junior □Senior |
| --- |

二、There are 24 questions in this section, please put a "√" on the number that matches your most similar situation.

| Items | Disagree | Slightly agree | Comparative agree | Strongly agree |
| --- | --- | --- | --- | --- |
| 1.Using smartphone helps me relax when I'm on edge. | 1 | 2 | 3 | 4 |
| 2.When I am stressed, using smartphone can calm me down. | 1 | 2 | 3 | 4 |
| 3.When I am depressed, using smartphone can make me feel comforted. | 1 | 2 | 3 | 4 |
| 4.Using smartphone helps me relax. | 1 | 2 | 3 | 4 |
| 5.Using smartphone can help me release the pressure. | 1 | 2 | 3 | 4 |
| 6.Using smartphone can relieve my anxiety. | 1 | 2 | 3 | 4 |
| 7.When I feel lonely, using a smartphone can make me feel more social. | 1 | 2 | 3 | 4 |
| 8.Using smartphone can make me feel happy. | 1 | 2 | 3 | 4 |
| 9.Using smartphone helps me socialize. | 1 | 2 | 3 | 4 |
| 10.Using smartphone can alleviate my negative feelings. | 1 | 2 | 3 | 4 |
| 11.Using smartphone can reduce my annoyance. | 1 | 2 | 3 | 4 |
| 12.Using smartphone makes me more tolerant of anxiety. | 1 | 2 | 3 | 4 |
| 13.Using smartphone helps control my negative thoughts. | 1 | 2 | 3 | 4 |
| 14.Using smartphone can distract my attention from the problem. | 1 | 2 | 3 | 4 |
| 15.I can't control my use of my smartphone. | 1 | 2 | 3 | 4 |
| 16.No matter how restrained I am, I still use my smartphone. | 1 | 2 | 3 | 4 |
| 17.Smartphone use controls my life. | 1 | 2 | 3 | 4 |
| 18.Even though I think it would be better to stop using my smartphone, I continue to use it. | 1 | 2 | 3 | 4 |
| 19.I have no control over how much time I use my smartphone. | 1 | 2 | 3 | 4 |
| 20.When I use my smartphone, I forget everything. | 1 | 2 | 3 | 4 |
| 21.For me, smartphone use is becoming an addictive behavior. | 1 | 2 | 3 | 4 |
| 22.The use of smartphone has affected my daily life. | 1 | 2 | 3 | 4 |
| 23.If I can't control my smartphone use, I will lose myself. | 1 | 2 | 3 | 4 |
| 24.Using smartphone is bad for my mind. | 1 | 2 | 3 | 4 |

三、There are 39 questions in this section, please put a "√" on the number that matches your most similar situation.

| Items | Strongly disagree | Comparative disagree | Not sure | Comparative agree | Strongly agree |
| --- | --- | --- | --- | --- | --- |
| 1.My classmates and friends often say that I spend too much time on my phone. | 1 | 2 | 3 | 4 | 5 |
| 2.I feel the need to spend more time on my phones to be satisfied. | 1 | 2 | 3 | 4 | 5 |
| 3.One of the direct results of spending time on playing mobile phone is that my study efficiency is reduced. | 1 | 2 | 3 | 4 | 5 |
| 4.Friends and family complain that I use my smartphone too much. | 1 | 2 | 3 | 4 | 5 |
| 5.I'd rather chat on my mobile phone than have a face-to-face conversation. | 1 | 2 | 3 | 4 | 5 |
| 6.When I am sad, the first thing I think of is playing smartphone. | 1 | 2 | 3 | 4 | 5 |
| 7.If I don't have my phone handy for a while, I often worry about missing the call. | 1 | 2 | 3 | 4 | 5 |
| 8.I had to open the same mobile app more than three times in the same day. | 1 | 2 | 3 | 4 | 5 |
| 9.Playing smartphone affects my academic performance. | 1 | 2 | 3 | 4 | 5 |
| 10.The procrastination caused by playing with my smartphone has caused me a lot of trouble. | 1 | 2 | 3 | 4 | 5 |
| 11.I get anxious if I can't use my phone for a while. | 1 | 2 | 3 | 4 | 5 |
| 12.I keep track of the latest apps and download them to my mobile phone. | 1 | 2 | 3 | 4 | 5 |
| 13.My mobile phone is an important part of my life, and when I reduce it, I feel like I'm losing something. | 1 | 2 | 3 | 4 | 5 |
| 14.When my mobile phone doesn't ring for a while, I feel uncomfortable and subconsciously check my phone for missed calls and text messages. | 1 | 2 | 3 | 4 | 5 |
| 15.When my mobile phone doesn't connect and I can't get a signal, I get anxious and my temper gets cranky. | 1 | 2 | 3 | 4 | 5 |
| 16.I find it hard to sleep because I use my smartphone to check what my friends are doing online. | 1 | 2 | 3 | 4 | 5 |
| 17.My academic performance has dropped because of playing smartphone. | 1 | 2 | 3 | 4 | 5 |
| 18.I unconsciously open certain apps on my mobile phone. | 1 | 2 | 3 | 4 | 5 |
| 19.I often have "my mobile phone is ringing/my mobile phone is vibrating" hallucinations. | 1 | 2 | 3 | 4 | 5 |
| 20.I always care about updating the apps I already have on my smartphone and keeping them up to date. | 1 | 2 | 3 | 4 | 5 |
| 21.I'm restless without my mobile phone. | 1 | 2 | 3 | 4 | 5 |
| 22.When I run out of things to say to my friends, I open the web app on my mobile phone. | 1 | 2 | 3 | 4 | 5 |
| 23.I worry that other people have had more rewarding experiences than I have. | 1 | 2 | 3 | 4 | 5 |
| 24.I am worried that my friend has more rewarding experiences than I do. | 1 | 2 | 3 | 4 | 5 |
| 25.I get worried when I find my friends happy without me. | 1 | 2 | 3 | 4 | 5 |
| 26.I get anxious when I don't know what my friends are doing. | 1 | 2 | 3 | 4 | 5 |
| 27.I always stay online so as not to miss anything. | 1 | 2 | 3 | 4 | 5 |
| 28.It's important that I have a say in the latest issues in my social network (e.g. videos, pictures, posts, etc.). | 1 | 2 | 3 | 4 | 5 |
| 29.I'm afraid I'm not the most fashionable on social networking sites. | 1 | 2 | 3 | 4 | 5 |
| 30.I kept scrolling through my smartphone so as not to miss anything. | 1 | 2 | 3 | 4 | 5 |
| 31.When I'm having a good time, it's important for me to share happy details online (like updating my status). | 1 | 2 | 3 | 4 | 5 |
| 32.It's important to know the Internet slang my friends use. | 1 | 2 | 3 | 4 | 5 |
| 33.When I take time off, I keep up with what my friends are doing. | 1 | 2 | 3 | 4 | 5 |
| 34.In situations of uncertainty, I always hope for the best. | 1 | 2 | 3 | 4 | 5 |
| 35.For me, if things can go wrong, they actually do. | 1 | 2 | 3 | 4 | 5 |
| 36.I am optimistic about my future. | 1 | 2 | 3 | 4 | 5 |
| 37.I never expect things to go the way I want them to. | 1 | 2 | 3 | 4 | 5 |
| 38.I never expect good things to happen to me. | 1 | 2 | 3 | 4 | 5 |
| 39.In general, I expect good things to happen to me rather than bad things. | 1 | 2 | 3 | 4 | 5 |
